# Hypertension is associated with greater heat exchange during exercise recovery in a hot environment

**DOI:** 10.1590/1414-431X20154532

**Published:** 2015-09-18

**Authors:** S. F. Fonseca, M. C. Teles, V. G. C. Ribeiro, F. C. Magalhães, V. A. Mendonça, M. F. D. Peixoto, L. H. R. Leite, C. C. Coimbra, A. C. R. Lacerda

**Affiliations:** 1 Centro Integrado de Pós-Graduação e Pesquisa em Saúde, Universidade Federal dos Vales do Jequitinhonha e Mucuri, Diamantina, MG, Brasil; 2 Programa Multicêntrico de Pós-Graduação em Ciências Fisiológicas, Sociedade Brasileira de Fisiologia, São Paulo, SP, Brasil; 3 Instituto de Ciências Biológicas, Universidade Federal de Juiz de Fora, Juiz de Fora, MG, Brasil; 4 Instituto de Ciências Biológicas, Universidade Federal de Minas Gerais, Belo Horizonte, MG, Brasil

**Keywords:** Hypertension, Hot Environment, Thermoregulation

## Abstract

Individuals with systemic arterial hypertension have a higher risk of heat-related complications. Thus, the aim of this study was to examine the thermoregulatory responses of hypertensive subjects during recovery from moderate-intensity exercise performed in the heat. A total of eight essential hypertensive (H) and eight normotensive (N) male subjects (age=46.5±1.3 and 45.6±1.4 years, body mass index=25.8±0.8 and 25.6±0.6 kg/m^2^, mean arterial pressure=98.0±2.8 and 86.0±2.3 mmHg, respectively) rested for 30 min, performed 1 h of treadmill exercise at 50% of maximal oxygen consumption, and rested for 1 h after exercise in an environmental chamber at 38°C and 60% relative humidity. Skin and core temperatures were measured to calculate heat exchange parameters. Mean arterial pressure was higher in the hypertensive than in the normotensive subjects throughout the experiment (P<0.05, unpaired *t*-test). The hypertensive subjects stored less heat (H=-24.23±3.99 W·m^−2^ *vs* N=-13.63±2.24 W·m^−2^, P=0.03, unpaired *t*-test), experienced greater variations in body temperature (H=-0.62±0.05°C *vs*N=-0.35±0.12°C, P=0.03, unpaired *t*-test), and had more evaporated sweat (H=-106.1±4.59 W·m^−2^ *vs* N=-91.15±3.24 W·m^−2^, P=0.01, unpaired *t*-test) than the normotensive subjects during the period of recovery from exercise. In conclusion, essential hypertensive subjects showed greater sweat evaporation and increased heat dissipation and body cooling relative to normotensive subjects during recovery from moderate-intensity exercise performed in hot conditions.

## Introduction

Exercise in a hot environment promotes physiological changes in the thermoregulatory, cardiovascular, and endocrine systems. These changes result in prolonged elevation of the core body temperature that persists during recovery from exercise (1). Previous studies using healthy individuals have demonstrated that a prolonged elevation in core body temperature occurs concurrently with a rapid decrease in sweating (2) and blood flow to the skin (3), and a decrease in skin temperature (2,3) to baseline values during the early stages of recovery.

Individuals with systemic arterial hypertension (SAH) are less able to adapt to extreme environmental conditions and have a higher risk of heat-related complications (4) than individuals with normal blood pressure (BP). This could be due to increased peripheral vascular resistance and accompanying peripheral circulatory changes (5), such as hypertrophy of the vascular smooth muscles (6), fewer blood vessels (7), and reduced sensitivity of the baroreflex in hypertensive individuals (8). Each of these changes could impair the control of blood flow to the skin (9) and consequently alter core temperature regulation. During exercise performed in hot conditions, hypertensive subjects experience an elevated BP response and possibly greater thermal strain than normotensive subjects (10).

It has previously been shown (10,11) that the increase in forearm blood flow that occurs during exercise-induced hyperthermia, which is primarily confined to the skin (12), is markedly lower in hypertensive individuals. This could lead to a reduction in heat transfer from the body core to the skin, thereby increasing the potential for heat-related illness (4). However, some studies have suggested that the thermoregulatory responses to passive exposure to a hot environment do not differ between subjects with and without hypertension (5). No differences were found in core temperature, heat exchange, cardiac work, sweat rate (10,11,13), and skin temperature (10,11) between hypertensive and normotensive individuals during physical exercise in the heat. However, these studies did not investigate the thermoregulatory responses of hypertensive individuals during the recovery period after exercise performed in the heat, a period during which heat dissipation is critical to return the body temperature to baseline values and avoid thermal damage.

Moreover, studies in healthy subjects have shown that an increase in the magnitude of post-exercise hypotension appears to be associated with an increased threshold for cutaneous vasodilation and sweating (14). This is a consequence of baroreceptor unloading due to lower body venous blood pooling (14). Once sweat evaporation is directly influenced by cutaneous vasodilation and sweating, any increase in the threshold for these may reduce heat loss and thus prolong elevations in core body temperature (15,16).

Hypertensive individuals have a marked hypotensive response during post-exercise recovery, which may be associated with central resetting of the baroreflex via discrete receptor changes within the nucleus tractus solitarii (17). Therefore, they may exhibit reduced ability to dissipate heat and consequently may cool their bodies less efficiently than normotensive individuals during recovery from moderate-intensity exercise performed under heat stress conditions. Thus, this study aimed to evaluate the thermoregulatory responses of both hypertensive and normotensive humans during recovery from moderate exercise performed in a hot environment.

## Material and Methods

### Ethical statement

This study was conducted in accordance with the ethical principles for research involving humans (Resolution #196-96 of the National Health Council of the Brazilian Ministry of Health) and received approval from the Ethics Committee of the Universidade Federal dos Vales do Jequitinhonha e Mucuri (protocol #024/12). All subjects were informed of the study procedures and provided their written consent to participate.

### Subjects

The study subjects were eight men with essential hypertension (H) and eight normotensive men (N), matched according to age, weight, height, and aerobic fitness.

### Procedures

A preliminary assessment was performed to determine the body composition of the subjects. This assessment of body composition was based on skin fold measurements (18), which were used to estimate body fat percentage (19). Body mass index (BMI) was calculated by measuring weight and height and applying Quetelet’s equation (20).

The BP of all subjects was measured on five consecutive mornings in their home after 10 min of rest in a sitting position using a mercury manometer with an inflatable cuff and a stethoscope (CJ0616, BIC, Brazil). The mean value of the five consecutive measurements for each participant was considered their baseline BP. Systolic and diastolic pressures were recorded as the first and the fourth Korotkoff sounds, respectively. The subjects were then evaluated by a cardiologist and underwent a maximum exercise stress test with a concurrent electrocardiogram. A progressive treadmill test (Inbramed Master CI, Brazil) was used to determine maximal oxygen consumption (VO2max). The protocol began with 2 min of walking at 3 km/h on a 5% slope; the pace was then increased by 1 km/h every 2 min while the slope was maintained at 5% throughout the entire test (10). Oxygen consumption was measured by an open circuit technique using a portable gas analyzer (K4, Italy) calibrated according to the manufacturer's recommendations. Data were collected breath-by-breath.

### Experimental conditions

The experimental procedures were performed in an environmental chamber with a dry-bulb temperature of 38°C and a relative humidity (RH) of 60%. Subjects wore shorts and rubber-soled shoes during the procedure. Each subject rested for 30 min in a sitting position and then completed 1 h of moderate-intensity treadmill exercise (50% VO2max, which represented approximately 80% of the ventilatory threshold for both groups). The treadmill was kept at a 5% slope during the entire protocol. The recovery phase was considered to start immediately after the exercise; the subjects sat at rest in the environmental chamber for the entire 1-h recovery period.

Core body temperature was monitored with a rectal probe (E-Val Flex, Denmark) coupled to a digital display; rectal temperature was recorded every 10 min throughout the experiment. Skin temperature was also monitored every 10 min using surface sensors (E-Val Flex) coupled to the digital display and positioned at four locations: leg, thigh, chest, and arm. To measure the oxygen consumption, a portable gas analyzer (K4b2, Cosmed, Italy) was used to collect breath-by-breath data. Heart rate (HR) was recorded every 5 min by a telemetric HR monitor (RS800sd, Polar, Brazil) and BP was recorded every 15 min throughout the experimental procedure using a mercury sphygmomanometer with an inflatable cuff and a stethoscope. The systolic and diastolic pressures were recorded as the pressures at the first and fourth Korotkoff sounds, respectively.

Body weight was measured immediately after physical exercise and again at the end of the recovery period to assess whole body sweat rates during post-exercise recovery in a hot environment.

### Hydration status

To ensure adequate hydration, subjects were instructed to drink 500 mL of water 2 h before the beginning of the experimental protocol. Urine specific gravity (Ug) was assessed as a measure of hydrations status 5 min before starting and 5 min after completion of the experimental protocol using a portable hand-held refractometer (model 301, Biobrix, Brazil) calibrated with distilled water. Subjects were considered hydrated when the Ug<1.030 (21).

During the experiment, the subjects drank a total of 600 mL of room temperature water (three portions of 200 mL each). The portions were offered at 3 specific times; the first after the initial 30 min of rest, the second immediately after treadmill exercise, and the third at the end of the recovery period.

### Calculations

Mean skin temperature (T_sk_, °C) was determined by the equation: T_sk_=0.3(TC+TA)+0.2 (TT+TL) where TC, TA, TT and TL are the temperatures of the chest, arm, thigh, and leg, respectively (22). Mean body temperature (T_b_, °C) was determined by the equation: T_b_=(0.2×T_sk_)+(0.8×core temperature) (23). To determine the mean change in body temperature (ΔT_b_) during each phase of the experiment (at rest, during exercise, and during the recovery period), the corresponding end point value was subtracted from the starting value for each phase.

Thermodynamic equations were used to calculate the heat exchange parameters at rest, and during and after moderate-intensity exercise. The convective heat transfer coefficient (h_c_, W·m^−2^·K^−1^) was calculated as h_c_=8.3×(v_a_^0.6^), where v_a_ is the air velocity (m·s^−1^) (24). The evaporative heat transfer coefficient (h_e_, W·m^−2^·kPa^−1^) was calculated as h_e_=16.5×h_c_ (24). Body surface area (A_D_, m^2^) was calculated as 0.00718×wt^0.425^×H^0.725^, where wt is body mass (kg) and H is height (cm) (25). The saturated water vapor pressure at the skin surface (P_s_, mmHg) was calculated as P_s_=1.92×T_sk_-25.3 (26). The energy equivalent (EE) of oxygen (J·LO_2_^−1^) was calculated as EE=(0.23×RER+0.77)×21166, where RER is the respiratory exchange ratio and 21166 is the energy equivalent of oxygen (O_2_, J·L^−1^) [modified from Parsons (27)]. The metabolic free energy production [M (W·m^−2^)=((EE×VO_2_×t)/(tx60))/A_D_], where VO_2_ is oxygen consumption (L·min^−1^), t is exercise time (min) and A_D_ is the body surface area (m^2^) [modified from Parsons (27)]. External work rate (W, W·m^−2^) was calculated as M ((wt×treadmill velocity×treadmill grade)/6.12)×A_D_, where treadmill velocity is in m·min^−1^, treadmill grade is expressed as a percent and 6.12 is the factor for conversion from kg·m·min^−1^ to watts. Internal heat production (H, W·m^2^) was calculated as H=M-W, where M is the free metabolic energy production and W is the external work rate (27). The heat storage rate (S), heat exchange by radiation (R, W·m^−2^) and convection (C) were calculated as described by Parsons (27): S=(3480×wt×ΔT_b_/t)/A_D_, where 3480 is the specific heat of body tissues (J·kg^−1^·°C^−1^) and t is time in s; R=((T_sk_-T_ra_)×5.2)/A_D_; and C=((T_sk_-T_a_)×v_a_^0.5^×8.3)/A_D_, where T_ra_ is the mean radiant temperature of the environmental chamber walls, T_a_ is the ambient temperature, 5.2 and 8.3 (W·m^−2^·°C^−1^) are the coefficients of heat exchange by radiation and convection, respectively, and T_sk_ is the mean skin temperature. The required evaporation rate (E_req_, W·m^−2^) was calculated as the residual component of: E_req_=H±R±C±S. The air velocity inside the environmental chamber was measured by a digital hot-wire anemometer (TAFR-180, Brazil) and remained constant (0.2 m/s) throughout the measurement.

The evaporative heat transfer via skin diffusion (E_d_, W·m^−2^) was calculated as E_d_=λ×m×(P_s_-P_a_), where λ is the latent heat of evaporation of sweat (2430 J·g^−1^), m is the permeance coefficient of the skin (1694×10^−4^g·s^−1^·m^−2^·mmHg^−1^), P_s_ is the partial water vapor pressure at the skin surface (mmHg), P_a_ is the partial water vapor pressure in ambient air (mmHg), and A_D_ is the body surface area (m^2^) modified from Fanger (26). The whole body sweat rate (M_sw_, W·m^−2^) was calculated as M_sw_={[(wt_initial_-wt_final_)-(fluid/food intake+urine/feces loss)-(0.019×VO_2_×(44-P_a_))×t]×2430}/[(t×60)×A_D_] where wt is body mass (g), fluid/food intake and urine/feces loss are in g, the expression 0.019×VO_2_×(44-P_a_) accounts for respiratory weight loss in g·min^−1^ (28), VO_2_ is oxygen uptake in L·min^−1^, and t is observation time (min). The heat transfer via evaporation and convection from the respiratory tract (E_res_+C_res_, W·m^−2^) was calculated as E_res_+C_res_=(0.0014×M×(T_ex_-T_db_))+(0.0017×M×(58.7-P_a_)), where M is the metabolic heat production (W·m^−2^), T_ex_ is expired air temperature (assumed to be 34°C), T_db_ is the dry bulb temperature (°C), and P_a_ is the partial water vapor pressure of ambient air (mmHg). The evaporated sweat (E_sw_, W·m^−2^) was calculated as E_sw_=E_req_-(E_d_+E_res_+C_res_).

### Statistical analysis

Prism software version 5.0 (USA) was used for the statistical analyses. The data are reported as means±SD. After testing the normality of the data by the Shapiro-Wilk test, Student's unpaired *t*-tests were used to assess between-group mean differences in weight, height, age, BMI, body fat percentage, body surface area, fitness, body temperature, heat storage, heat production, heat exchange parameters, whole body sweat rate, blood pressure, magnitude of hypotension, heart rate, and urine specific gravity. Furthermore, two-way ANOVA with Bonferroni *post hoc* testing was used to assess the significance of the interactions between group and time for the rectal and skin temperatures. After testing the sphericity of the data by means of the Mauchly test, repeated measures ANOVA was used to assess the significance of between-group differences in mean arterial pressure. It was not necessary to correct the degrees of freedom of the F-distribution (correction) because sphericity was not violated. The statistical power was calculated from the results, taking into consideration the effect sizes of the values obtained from comparisons between hypertensive and normotensive subjects during exercise recovery (29). Pearson’s correlation coefficients were used to assess the correlation between variables. The correlation was used to evaluate the direction and degree of the linear relationship between heat exchange by evaporation and the rate of heat storage, as well as between heat exchange by evaporation and the change in mean body temperature during the recovery period. The significance level for all tests was 5% (P<0.05).

## Results

There were no between-group differences in weight, height, age, BMI, body fat percentage, body surface area, and fitness; however, MAP was higher in the hypertensive group. All of the hypertensive individuals were currently taking a combination of diuretics and angiotensin-converting enzyme (ACE) inhibitors at the time of the study (Table 1).

**Table:**
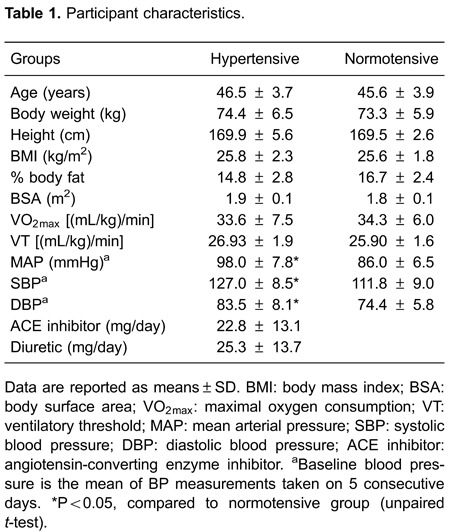

**Figure 1:**
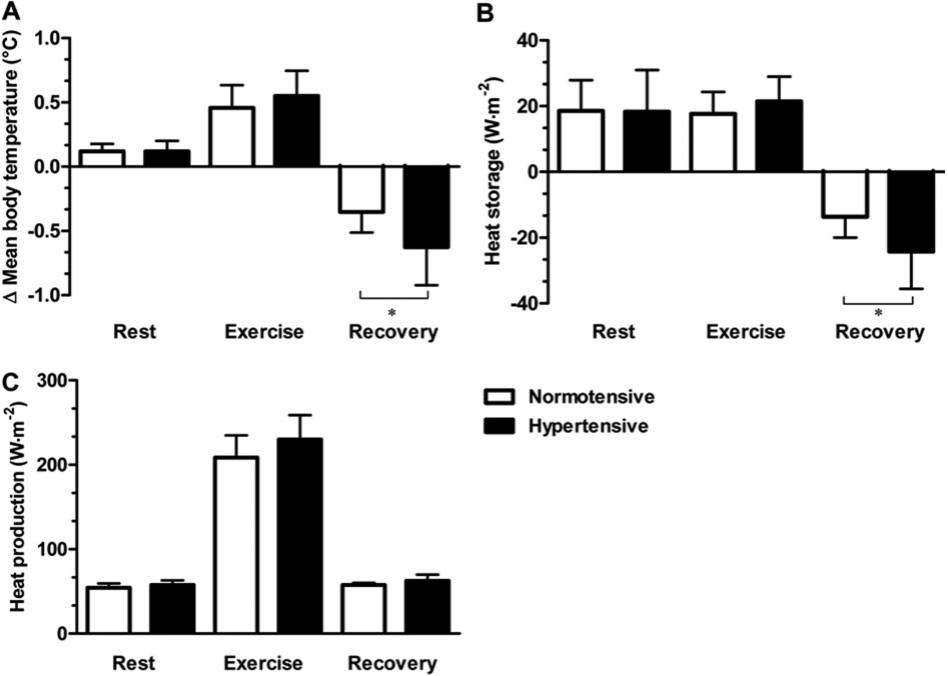
Thermoregulatory parameters during the experiment. The change in the average body temperature (*A*), heat storage rate (*B*), and heat production (*C*) of normotensive (n=8) and hypertensive (n=8) subjects at rest, during, and after physical exercise in a hot environment (38°C and 60% relative humidity). Data are reported as means±SD. *P<0.05 hypertensive compared to normotensive (unpaired *t*-test). The baseline average body temperatures of hypertensive and normotensive subjects were 35.96±0.23°C and 35.83±0.22°C, respectively.

As shown in Figure 1, there were no differences between the hypertensive and normotensive subjects in mean body temperature variation, heat storage, and heat production at rest and during exercise. However, during the post-exercise recovery there was a greater reduction in mean body temperature (A, P=0.03) as well as less heat storage (B, P=0.03) in the hypertensive group relative to the normotensive group.

**Figure 2:**
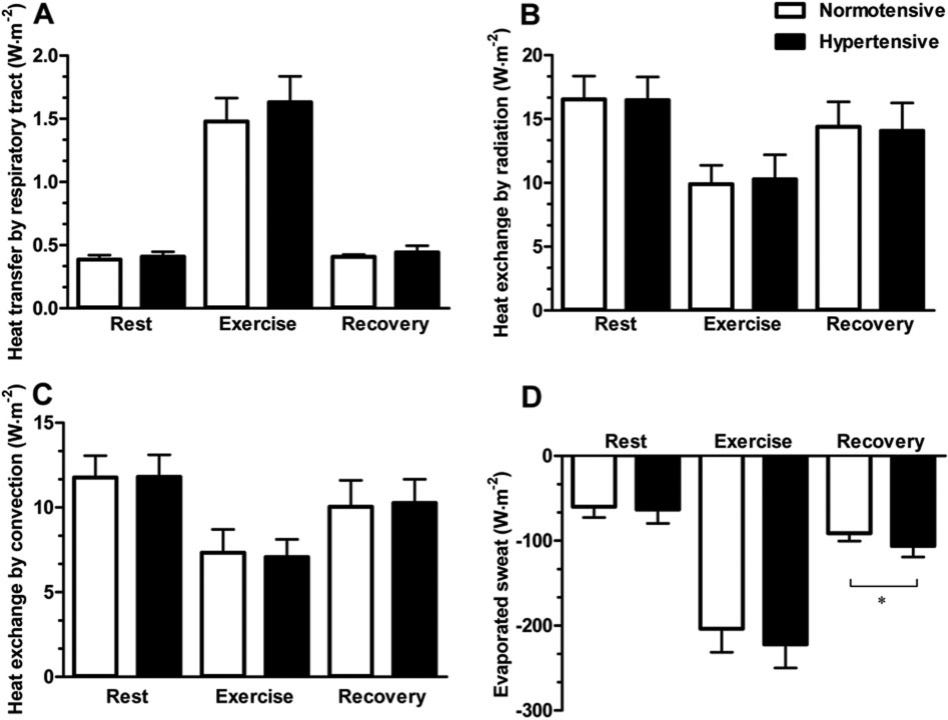
Heat exchange parameters calculated during the experiment. Transfer of heat by the respiratory tract (A) and heat exchange by: radiation (B), convection (C) and sweat evaporation (D) in normotensive (n=8) and hypertensive (n=8) subjects at rest, during, and after physical exercise in a hot environment (38°C and 60% relative humidity). Data are reported as means±SD. *P<0.05 hypertensive compared to normotensive (unpaired t-test).

Figure 2 shows the results of analysis of the thermodynamic parameters. At rest, the transfer of heat by the respiratory tract (A, P=0.23) and heat exchange by radiation (B, P=0.95), convection (C, P=0.95), and the evaporation of sweat (D, P=0.65) were not different between groups. During physical exercise, there was a similar increase in the transfer of heat by the respiratory tract (A, P=0.144) and in the heat exchange by radiation (B, P=0.67), convection (C, P=0.67), and evaporation (D, P=0.26). During the exercise recovery period, individuals with hypertension exchanged more heat by sweat evaporation than normotensive individuals (D, P=0.01). Moreover, heat exchange by radiation and convection (B and C, P=0.76) and heat transfer by the respiratory tract (A, P=0.09) were similar between groups during exercise recovery. The calculated statistical power values for the rate of heat storage, heat exchange by evaporation, and the change in the average body temperature during exercise recovery, considering an effect size of 1.1, 1.3, and 1.1, were 68%, 80%, and 68%, respectively (alpha value=0.5 and n=8).

**Figure 3:**
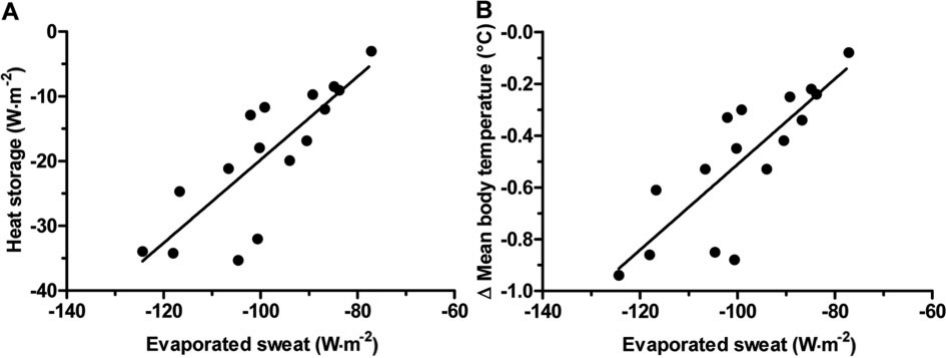
Correlation of evaporated sweat with heat storage (*A*) and change in mean body temperature (*B*). *A*, P<0.001, r=0.82, R^2^=0.68; *B*, P<0.001, r=0.82, R^2^=0.67 (Pearson’s correlation).

Body weight was similar between the groups immediately after exercise (H=74.64±6.3 kg, N=73.79±5.8 kg; P=0.79) and at the end of the recovery period (H=74.45±6.3 kg, N=73.68±5.8 kg; P=0.80). However, the whole body sweat rate was significantly higher in hypertensive subjects compared to normotensive subjects during the post-exercise recovery period (H=132.4±21.2 W·m^−2^, N=78.5±8.8 W·m^−2^; P=0.03). Figure 3 shows the correlation between heat exchange by evaporation and the rate of heat storage (A) (r=0.82, r^2^=0.68, P<0.0001) and between heat exchange by evaporation and the change in average body temperature (B) (r=0.82, r^2^=0.67, P<0.0001) during the recovery period.

**Figure 4:**
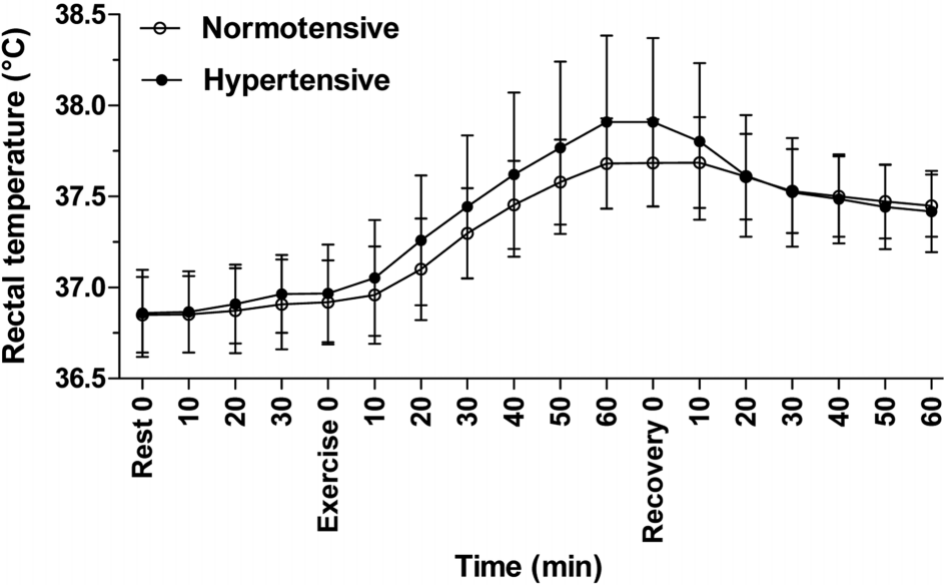
Core body (rectal) temperature (°C) during the experiment. Data were collected from normotensive (n=8) and hypertensive (n=8) subjects every 10 min at rest, during, and after physical exercise in a hot environment (38°C and 60% relative humidity). Data are reported as means±SD (two-way ANOVA).

**Figure 5:**
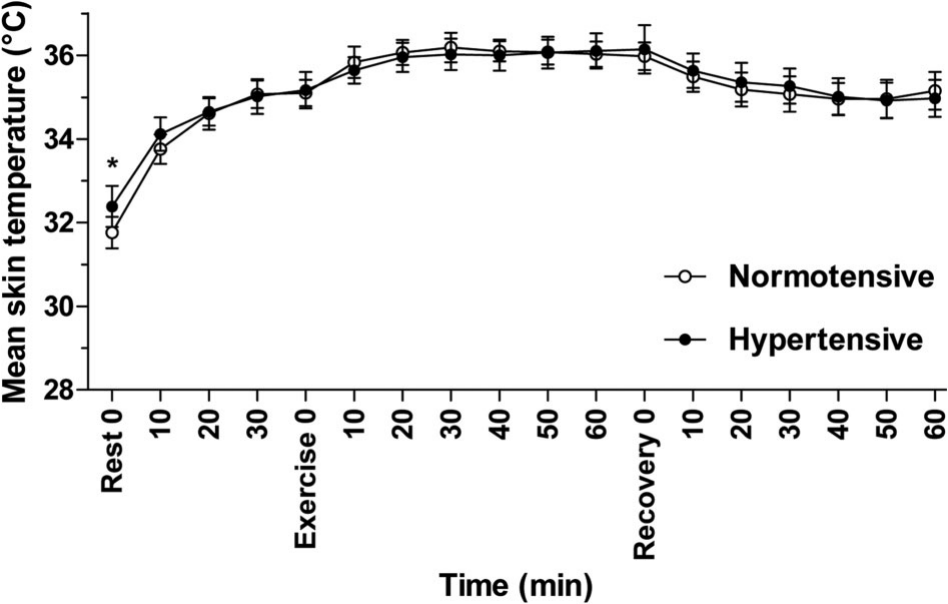
Skin temperature (°C) during the experiment. Data were collected from normotensive (n=8) and hypertensive (n=8) subjects every 10 min at rest, during, and after physical exercise in a hot environment (38°C and 60% relative humidity). Data are reported as means±SD. *P<0.05 hypertensive compared to normotensive (two-way ANOVA).

There was no significant difference in the mean core body (rectal) temperatures of hypertensive and normotensive individuals throughout the experiment (P=0.54; Figure 4). Mean skin temperature differed significantly between the groups only at the beginning of the initial rest period before exercise, and then remained similar between the groups throughout the experimental protocol (P=0.72; Figure 5).

**Figure 6:**
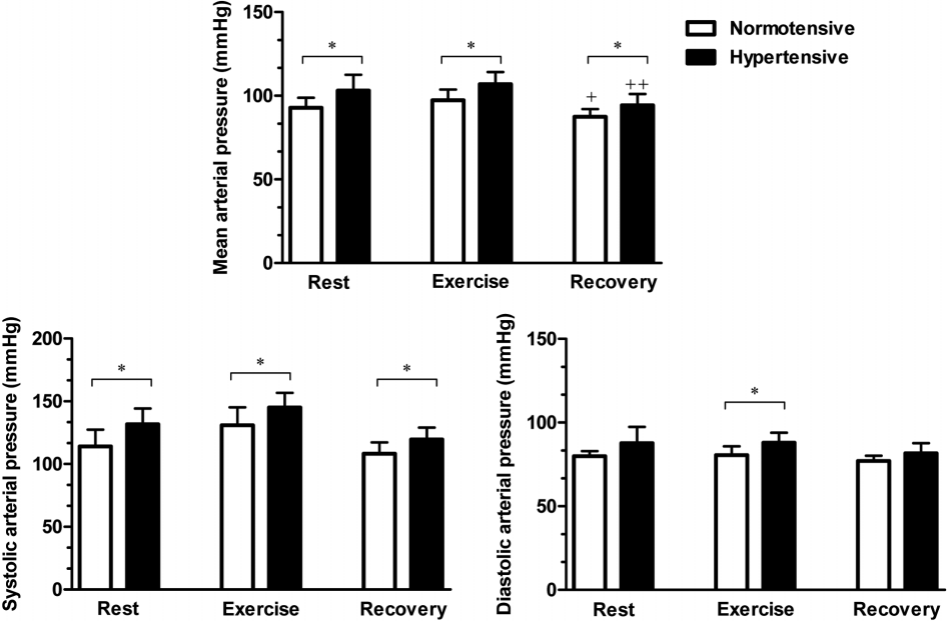
Mean arterial pressure, and systolic and diastolic arterial pressures during the experiment. Data are reported as means±SD. *P<0.05 hypertensive compared with normotensive (unpaired *t*-test). ^+^P<0.001 compared with normotensive rest value (repeated measures ANOVA). ^++^P<0.001 compared with hypertensive rest value (repeated measures ANOVA). The magnitude of the post-exercise hypotension was 8.7±1.8 mmHg in hypertensive subjects and 5.4±1.1 mmHg in normotensive subjects (P=0.14).

Systolic arterial pressure was higher in the hypertensive group than in the normotensive group throughout the experiment (P<0.05), while diastolic arterial pressure was higher in the hypertensive group only during exercise (P<0.05). Similarly, MAP was higher in the hypertensive group than in the normotensive group throughout the experiment (P<0.05). There was post-exercise hypotension in both the normotensive (P<0.001) and hypertensive (P<0.001) groups (Figure 6). However, the magnitude of the post-exercise hypotension did not differ between the groups (H=8.7±1.8 mmHg, N=5.4±1.1 mmHg, P=0.14).

HR did not differ between the groups throughout the experimental protocol (rest: H=79.1±3.13 bpm, N=73.4±3.05 bpm, P=0.22; exercise: H=121.5±6.1 bpm, N=118.5±3.74 bpm, P=0.67; recovery H=100.3±6.9 bpm, N=94.6±7.92 bpm, P=0.59). All subjects were considered hydrated, and urine specific gravity did not differ between groups before (H=1015±2:06 Ug, N=1011±1.99 Ug, P=0.28), or at the end (H=1013±1.78 Ug, N=1011±1.19 Ug, P=0.37) of the experiment.

## Discussion

The results of the present study indicate that, compared with normotensive individuals, hypertensive individuals lose more heat through sweat evaporation during recovery from moderate-intensity treadmill exercise in a hot environment. During exercise recovery, hypertensive individuals experience a greater reduction in both mean body temperature variation (Δ) and in heat storage that correlates with heat exchange by evaporation (Figures 3A and B) than normotensive individuals, which suggests that the greater heat exchange in hypertensive subjects is due to a greater whole body sweat rate and a higher rate of sweat evaporation.

Given that 1) some organizations have warned about climate change on Earth with the prospect of further increases in both the average global temperature and the frequency of heat waves (30) and 2) moderate-intensity exercise training is widely prescribed as a tool for social outreach and nonpharmacological treatment of hypertension (31) especially in subjects with mild (grade 2) essential hypertension, it is important to understand the interactions between environmental heat stress and physical exercise in terms of the thermoregulatory response during recovery from physical exercise in hot conditions. To the best of our knowledge, this is the first study investigating the thermoregulatory responses of hypertensive individuals during recovery from moderate-intensity exercise performed in a hot environment. Previous studies (2,) have demonstrated that post-exercise temperature regulation can be modulated by baroreceptor unloading, which may potentially differ between hypertensive and normotensive individuals. However, both groups in this study experienced a similar post-exercise hypotensive response; thus, the hypothesis that the hypertensive group had a marked hypotensive response that was associated with central resetting of the baroreflex and a reduced response to heat dissipation during recovery from exercise in the heat, does not seem plausible.

It has been previously shown (10,11,13) that hypertensive subjects tolerate hot conditions similarly to normotensive subjects when performing 60 min of submaximal exercise at 40% VO2max in a hot environment. Previous studies have found no between-group differences in core body temperature, calculated heat exchange variables, sweat rate (10,11,13), and skin temperature (10,11). Furthermore, Kenney et al. (10) demonstrated that forearm blood flow during exercise-induced hyperthermia is markedly lower in hypertensive subjects compared with that in normotensive subjects. However, these studies did not evaluate thermodynamic parameters during post-exercise recovery, which is a phase that requires integrated physiological responses to restore thermal equilibrium and return core body temperature to baseline levels. To the best of our knowledge, the present study is the first to demonstrate increases in sweat evaporation and sweat rate in hypertensive subjects during exercise recovery. These responses are associated with greater body cooling during recovery from exercise in a hot environment in hypertensive subjects compared with those of normotensive subjects. These findings suggest that increased cholinergic sympathetic activation of sweating (32,33) influences the rate of sweating and sweat evaporation in hypertensive subjects. Although increased sweating and sweat evaporation are mechanisms that explain the greater body cooling ability of hypertensive individuals during recovery from exercise in a hot environment, changes in blood flow to the skin and heat conductance may be mediated by cholinergic sympathetic nerves. However, Kellogg Jr. et al. (5) reported that the cutaneous vascular conductance of the forearm was similar in hypertensive and normotensive subjects during passive heat stress. In addition, during hyperthermia, the core temperature at which vasodilation began did not differ between groups.

Control of thermoregulation in the central nervous system occurs mainly in the hypothalamus, and previous studies have shown that cholinergic pathways are involved in this central control (34). Furthermore, studies using animal models have demonstrated that the central cholinergic system plays a role in increasing tail blood flow (34,35), and BP (36,37), reducing body temperature, and increasing heat dissipation during exercise (36,37) and post-exercise recovery (35). Thus, the information currently available suggests that hypertension may be associated with increased peripheral sympathetic stimulation dependent on central cholinergic activity (38,39). Our results are consistent with this, and support the hypothesis that activation of the central cholinergic system plays an important role in the modulation of sympathetic tone and in the activation of heat dissipation (3436,). Therefore, we propose that the increased activity of the central cholinergic system in individuals with essential hypertension may have increased sweating via sympathetic activation (32,33). Additionally, an increase in central cholinergic activity may reduce central serotonergic activity and this, in turn, could modulate the activity of the central dopaminergic pathway that inhibits sympathetic adrenergic stimulation of the peripheral vessels. Suppression of adrenergic sympathetic signaling may result in greater heat conduction to the periphery, with subsequent greater dissipation of heat, especially during recovery from moderate-intensity physical exercise.

The hypertensive subjects in this study had BP values that were higher than those of the normotensive subjects at the time of selection and throughout the experiment, despite treatment with antihypertensive medications; this indicates that these individuals had increased peripheral sympathetic drive at the time of the study. However, despite the increased BP observed in the hypertensive subjects, HR did not differ between the groups during any phase of the experiment (during rest, exercise, or recovery). This is likely because the hypertensive subjects in the present study did not have altered baroreceptor sensitivity.

Combining different medications in the treatment of hypertension is common in clinical practice. Because all of the hypertensive subjects were taking medication during the study, the additional effects of the drugs on peripheral vasodilation and, therefore sweat evaporation, cannot be neglected. It is possible that the combination of ACE inhibitors and diuretics caused greater cutaneous vasodilation in the hypertensive subjects during the recovery phase, allowing more blood flow to the skin and increasing sweat evaporation. The amount of sweat produced when blood reaches the periphery of the body could influence the evaporation of sweat during recovery from exercise performed in the heat in hypertensive subjects. Therefore, it is reasonable to suggest that the higher sweat production shown by hypertensive subjects may have increased their heat exchange by sweat evaporation. The result of this increased sweat evaporation was a greater reduction in mean body temperature and less heat storage. Further study of the relationship between antihypertensive drug actions and central cholinergic sympathetic activation in individuals with essential hypertension after moderate-intensity exercise performed in a hot environment will further our understanding of the physiological mechanisms underlying the results of this study.

In addition, the skin temperature of the hypertensive subjects was higher than that of the normotensive subjects when they were at rest in the environmental chamber prior to exercise (Figure 5). Thus, it is reasonable to suggest that the combination of ACE inhibitors and diuretics taken by the hypertensive subjects caused greater cutaneous vasodilation and consequently more blood flow to the skin and a higher skin temperature. However, because we did not monitor the environmental conditions that the volunteers were exposed to before entering the environmental chamber, we cannot definitively state whether the between-group difference in mean skin temperature at the beginning of the initial rest period was influenced by their medications or by the environmental conditions outside of the chamber.

Despite the relevance of this study, it had several limitations. Because the hypertensive subjects were taking antihypertensive medications (diuretics and ACE inhibitors) to control blood pressure, caution must be used when generalizing these results to other populations. In addition, the small sample size might also be considered a potential limitation of the present study. However, because differences in subject demographics (such as age, body surface area, and medication use) could produce variations in their thermoregulatory responses, we used only a small number of subjects to ensure that the groups were well matched. Finally, it is important that studies using different exercise intensities and/or heat stress, as well as other types of subjects (such as hypertensive without medication) are conducted to enhance our understanding of the effects of heat stress on thermoregulatory responses during recovery from exercise in individuals with hypertension.

In conclusion, the results of this study demonstrate that individuals with essential hypertension have a greater whole body sweat rate and increased sweat evaporation, and consequently they dissipate more heat and experience greater body cooling than normotensive individuals during recovery from moderate-intensity exercise performed in a hot environment.
